# Transvaginal hybrid-NOTES vs. traditional laparoscopic sigmoid resection for diverticulitis: a short-term comparative study

**DOI:** 10.1038/s41598-020-79461-1

**Published:** 2020-12-18

**Authors:** Marie Derstadt, Panagiotis Thomaidis, Claudia S. Seefeldt, Jonas Lange, Jurgen Meyer, Michael A. Ströhlein, Markus M. Heiss, Dirk R. Bulian

**Affiliations:** Department of Abdominal, Tumor, Transplant and Vascular Surgery, Cologne-Merheim Medical Center, Witten/Herdecke University, Ostmerheimer Strasse 200, 51109 Cologne, Germany

**Keywords:** Colonic diseases, Inflammatory bowel disease, Clinical trials

## Abstract

The aim was to compare short-term results of transvaginal hybrid-NOTES (NSR) with traditional laparoscopic technique in sigmoid resection (LSR) in cases of diverticulitis. Natural Orifice Transluminal Endoscopic Surgery has been evolved as a minimally invasive procedure to reduce the operative trauma due to the absence of specimen extraction through the abdominal wall causing less postoperative pain, and shorter hospital stay. Despite the increasing use and published case series of NSR for diverticulitis as a laparoscopic procedure with transvaginal stapling and specimen extraction, there are no studies comparing this procedure with LSR. Twenty NSR patients operated at the Cologne-Merheim Medical Center have been documented and compared with 20 female LSR patients matched for body mass index, American Society of Anesthesiologists-classification (ASA), Hansen/Stock classification, and age. To ensure comparability regarding peri- and postoperative care, only procedures performed by the same surgeon were included. Procedural time, intra- and postoperative complications, conversion rate, postoperative pain, the duration of an epidural catheter, analgesic consumption, and postoperative length of hospital stay were analyzed. There were no significant differences in the sum of pain levels (p = 0.930), length of procedure (p = 0.079), intra- and postoperative complications, as well as duration of an epidural catheter. On the contrary, there were significant positive effects for NSR on morphine requirement at day seven and eight (p = 0.019 and p = 0.035 respectively) as well as the postoperative length of hospital stay (p = 0.031). This retrospective study reveals significant positive effects for NSR compared to LSR regarding length of hospital stay as well as morphine consumption after removal of the epidural catheter, whereas there were no significant differences in complication rate and procedural time. In summary, NSR is an adequate alternative to traditional laparoscopic sigmoid resection considering the surgeons experience and the patient’s personal preferences.

## Introduction

The idea of using natural body orifices as an access route has existed since the beginning of the twentieth century. ***Dimitrij Oscarovic v. Ott (1903) was a pioneer with his “Ventroscopy”^1^. Until 2000, only a few publications on different access routes were available^2–4^. Since 2000, case descriptions and studies on animals and humans have been increasingly published^5^. The aim of this modification of minimally invasive surgery was to reduce the operative trauma and thereby the postoperative pain by reducing the number of abdominal access routes or the size of the required incisions in the abdominal wall especially by eliminating the need for minilaparotomy for specimen removal^6^. In addition to earlier mobilization of patients after surgery, a shorter hospital stay can also be seen as an advantage of less invasive procedures.

In Natural Orifice Transluminal Endoscopic Surgery (NOTES), all natural access routes to the abdominal cavity such as esophagus, stomach, rectum and in women the vagina can be used^7^.

In contrast to the other access routes, where only flexible endoscopes can be used, in a transvaginal access rigid instruments can be applied, which is more comfortable for most surgeons due to the familiar handling in laparoscopy. A further advantage for the transvaginal access is the long-term experience gained since the first transvaginal hysterectomy in the nineteenth century^8^. So, it is not surprising that especially in gynecology, but also in urology, this approach has also been used as a retrieval route for other intraperitoneal procedures and as an alternative access for e.g. intraoperative sonographic diagnostics^9,10^. In addition, the multidisciplinary approach is important to safely develop this procedure, which also harbors potential complications and requires surgical expertise and experience^11^.

The majority (97.9%) of NOTES procedures in general surgery performed in Germany until 2013 were transvaginal hybrid procedures, 87% of which were cholecystectomies, while bowel resections accounted for a significantly lower proportion (5.1%)^12^. In this group, sigmoid resections for diverticulitis were prevalent with 85%. So far, limited research has been conducted comparing the results of transvaginal hybrid NOTES sigmoid resection (NSR) to traditional laparoscopic technique (LSR).

The aim of our retrospective study was to investigate whether NSR is a viable alternative to LSR and if there are advantages or disadvantages regarding morbidity and mortality.

## Methods

### Patients

Since December 2008, more than 300 hybrid NOTES operations have been performed in the Department of Abdominal, Tumor, Transplant and Vascular Surgery of Cologne-Merheim Medical Center and were documented in a prospective database. In the beginning mainly cholecystectomies were performed, and subsequently also appendectomies as well as colorectal resections. A previous hysterectomy was not a contraindication for the transvaginal approach since the access was performed via the vaginal stump instead of the posterior vaginal vault. Since June 2012 the transvaginal hybrid NOTES sigmoid resection with rigid instruments has been offered as an alternative technique to the gold standard of traditional laparoscopic procedure in female patients requiring operative treatment for diverticulitis. Patients chose the surgical technique according to their own preferences. For this analysis, all NSR performed for diverticulitis until December 31, 2019 were extracted from the NOTES database and defined as the intervention group.

The comparison group was determined from all LSR performed for diverticulitis during the same period. In order to ensure good comparability only patients operated by D.R.B. were selected, due to the fact that all NSR were performed by him. Including the operations of other surgeons into the control group would lead to a systematic bias and significantly distort the results regarding intraoperative procedures and postoperative treatment. Furthermore, D.R.B. has years of experience in the surgical treatment of sigmoid diverticulitis, especially in laparoscopic technique. Taking the large experience in transvaginal approach together, we would state that the surgical experience of D.R.B. is equal for both investigated procedures. The 20 patients of the comparison group were selected on the basis of matched patient-specific parameters, meaning the American Society of Anesthesiologists-classification (ASA), age, body mass index (BMI) and diverticulitis classification according to Hansen/Stock.

**Figure 1:**
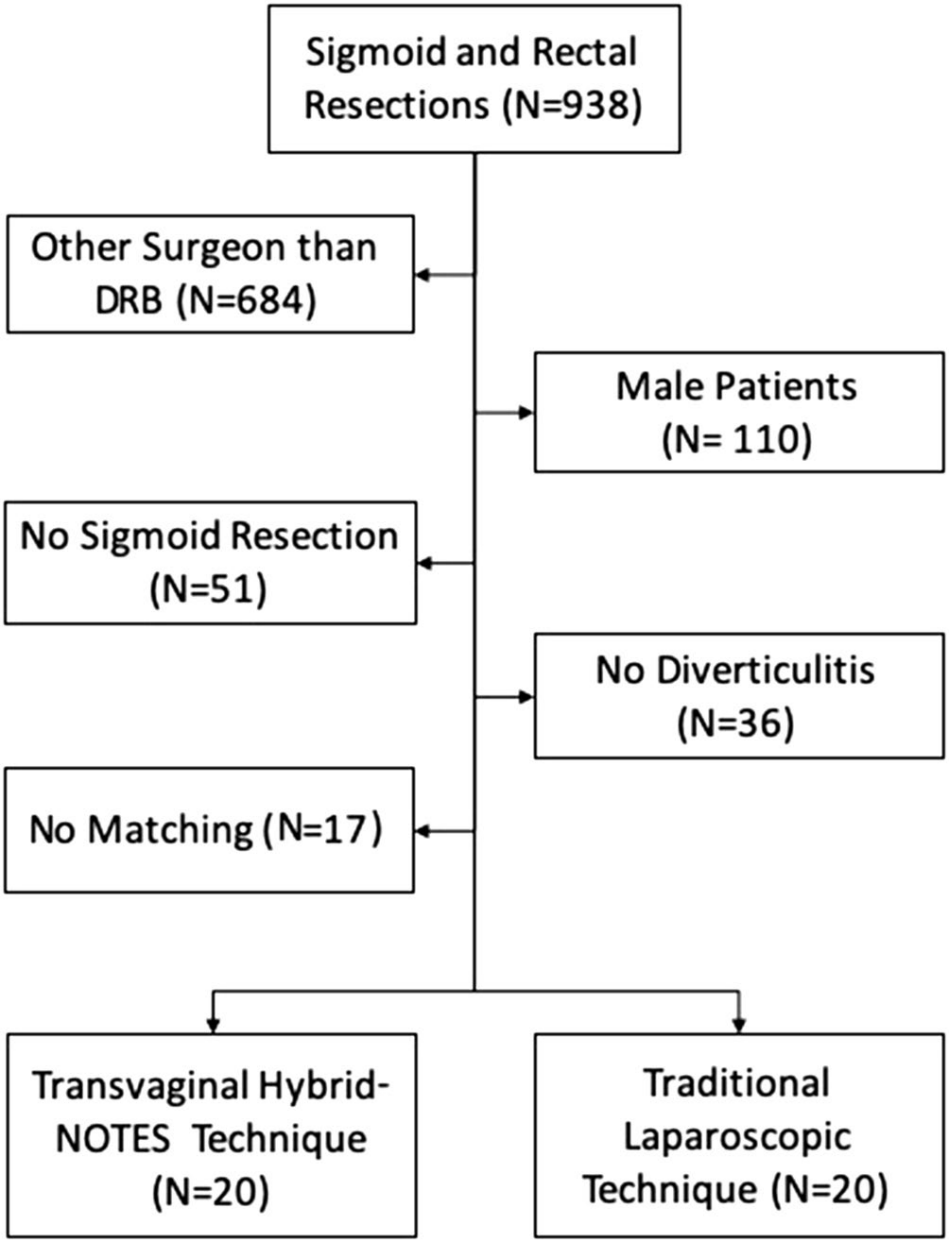
Trial flow diagram.

Since 2008 until 2019, 938 patients underwent sigmoid or rectal surgery in the Department of Abdominal, Tumor, Transplant and Vascular Surgery of Cologne-Merheim Medical Center. In 254 cases, the surgeries were performed by D.R.B. This criterion is used to prevent physician-specific differences from influencing the evaluation of the examined parameters. In total, 144 patients were female and 93 of them underwent sigmoid surgery—57 due to diverticulitis. Twenty cases of these were NSR. Of the remaining 37 LSR patients, 20 were matched with the NSR patients using the above-mentioned parameters: age, BMI, Hansen/Stock classification and ASA classification (Fig. 1).

### Surgical techniques

#### NSR

After establishing a capnoperitoneum up to 11 mmHg with a Veres needle, inserted subcostal in the left middle clavicular line, a 5 mm trocar is inserted via a supraumbilical, left paramedian access. Subsequently, three further 5 mm trocars are inserted after skin incisions on the left in the middle to upper abdomen far laterally, on the right laterocaudally of the navel as well as suprasymphysary. During the procedure, the left colonic flexure is completely mobilized from medial to lateral, the inferior mesenteric vein is sealed and cut through below the pancreas, and the left half of the greater omentum is detached from the transversal colon. After preparation and titanium clip closure, the inferior mesenteric artery can be cut through, and the sigmoid colon can be totally mobilized by loosening the embryonic and proinflammatory adhesions of the sigma. Both ureters are exposed and spared. Then the rectum is dissected up to the upper third. The oral and aboral resection lines are chosen depending on local post-inflammatory alterations and the mesocolon as well as the mesorectum are sealed in between. After exposure of the posterior vaginal vault by means of specula and anteflection of the uterus using a Sims’ uterus probe both under laparoscopic control and diaohanoscopy, a 13 mm trocar can be inserted. For patients with previous hysterectomy the vaginal stump was exposed and the trocar inserted above it under visual control. Via this access the Endo-GIA for the following closure and subsequent separation of both resection borders is inserted. After removal of the transvaginal trocar, a double ring wound retractor is inserted via this orifice. The specimen is then retrieved and delivered to histology. The anvil is inserted also transvaginally. The colpotomy or the vaginal stump is then closed with a running resorbable suture and the capnoperitoneum restored. After resection of the staple suture of the oral colonic stump the suture is placed in the open oral stump. Then the anvil can be knotted in the oral colonic stump by a purse-string suture, and the transanal end-to-end tension-free and rotationally correct double stapling anastomosis can be performed.

#### LSR

After a left supraumbilical skin incision, a 10 mm trocar is inserted in open technique and a capnoperitoneum up to 11 mmHg is established. Three 5 mm trocars are then inserted under visual control after respective diaphanoscopy and skin incision in the left lateral subcostal abdomen, right laterocaudal of the navel and suprasymphysary. The left half of the major omentum is detached from the transverse colon in layers and the left colonic flexure is completely mobilized as described in the NSR technique. After total mobilization of the sigmoid colon, the rectum is dissected and the mesorectum sealed up to the upper third aborally of the inflammatory change. Both ureters are also displayed and spared. Subsequently, the closure and separation of the aboral resection line is performed using a stapler after replacing the 10 mm trocar by a 13 mm trocar. The oral residual stump is clamped, and a limited Pfannenstiel incision is performed. After insertion of the wound retractor, the colon is luxated. The oral resection line in the descending colon is planned orally to the inflammatory changes, and the mesocolon is sealed and divided up to the centrally separated artery. The oral resection line is then sharply divided, and the specimen is delivered to histology. After the anvil has been knotted with a purse-string suture, the colon can be replaced. The capnoperitoneum is re-established after the ring foil has been removed and the retrieval incision has been closed in correct layers. Subsequently, a transanal end-to-end tension-free and rotationally correct double stapling anastomosis is performed.

An epidural catheter for analgesia was applied as standard to every patient who had no contraindication. As oral pain medication oxycodone and a non-steroidal anti-inflammatory drug (NSAID) were used.

### Outcome parameters

The analyzed intraoperative parameters were the duration of the operation in minutes, number of transabdominally inserted trocars, intraoperative complications and any necessary conversions of the surgical procedure. Conversion was defined in the NSR group as performing a traditional laparoscopic surgery with a retrieval incision or laparotomy or if transvaginal specimen retrieval was not possible. In the LSR group conversion was defined as performing a laparotomy.

To avoid bias, patients undergoing an additional surgical procedure simultaneously were excluded in the analysis of the length of surgery.

Postoperatively, the pain was assessed using the Numeric Rating Scale (NRS; Scale from 0 to 10: 0 = no pain, 10 = strongest pain imaginable). Other postoperative parameters including the duration of the epidural catheter in days, the need for analgesic medication, postoperative complication rate and their severity (using Clavien–Dindo classification)^13^, and length of hospital stay in days were assessed. In order to obtain comparability of the various opiate dosages, the morphine equivalent dose has been determined.

### Data collection

The data was retrieved by generating a query in the database of the hospital using the codes for sigmoid or rectal surgery of the German modification of the International Classification of Procedures in Medicine (ICPM). In addition, we retrieved the age of the patients at the time of the operation, the ASA classification, the duration of the operation and the surgeon of each procedure by this query. All other parameters were retrieved by searching through the medical records of the selected patients. The data collection was conducted by three team members.

### Statistical analysis

The data was prepared in Microsoft Excel and analyzed in SPSS Statistics Version 27 (IBM Corp., Armonk, NY, USA). Data of continuous variables are expressed as minimum, maximum and median. Binary and categorical variables are reported as counts and percentages. The Mann–Whitney U test was used for continuous parameters, the Chi-square test for categorical parameters. *p* < 0.05 was considered as statistically significant.

### Ethical standards

The study protocol was approved by our Institutional Review Board (Research Ethics Committee of Witten/Herdecke University) on March 17, 2016 (no. 01/2016) and meets the guidelines of the responsible governmental agency. Written and informed consent regarding the performed procedures as well as potential future analyses of their data was obtained from all patients.

## Results

In the period from 2008 to 2019 twenty NSR for diverticulitis were performed. The NSR group and the LSR group did not differ significantly in age (p = 0.569), BMI (p = 0.882), ASA classification (p = 0.598), and diverticulitis classification according to Hansen/Stock (p = 0.792).

**Figure 2:**
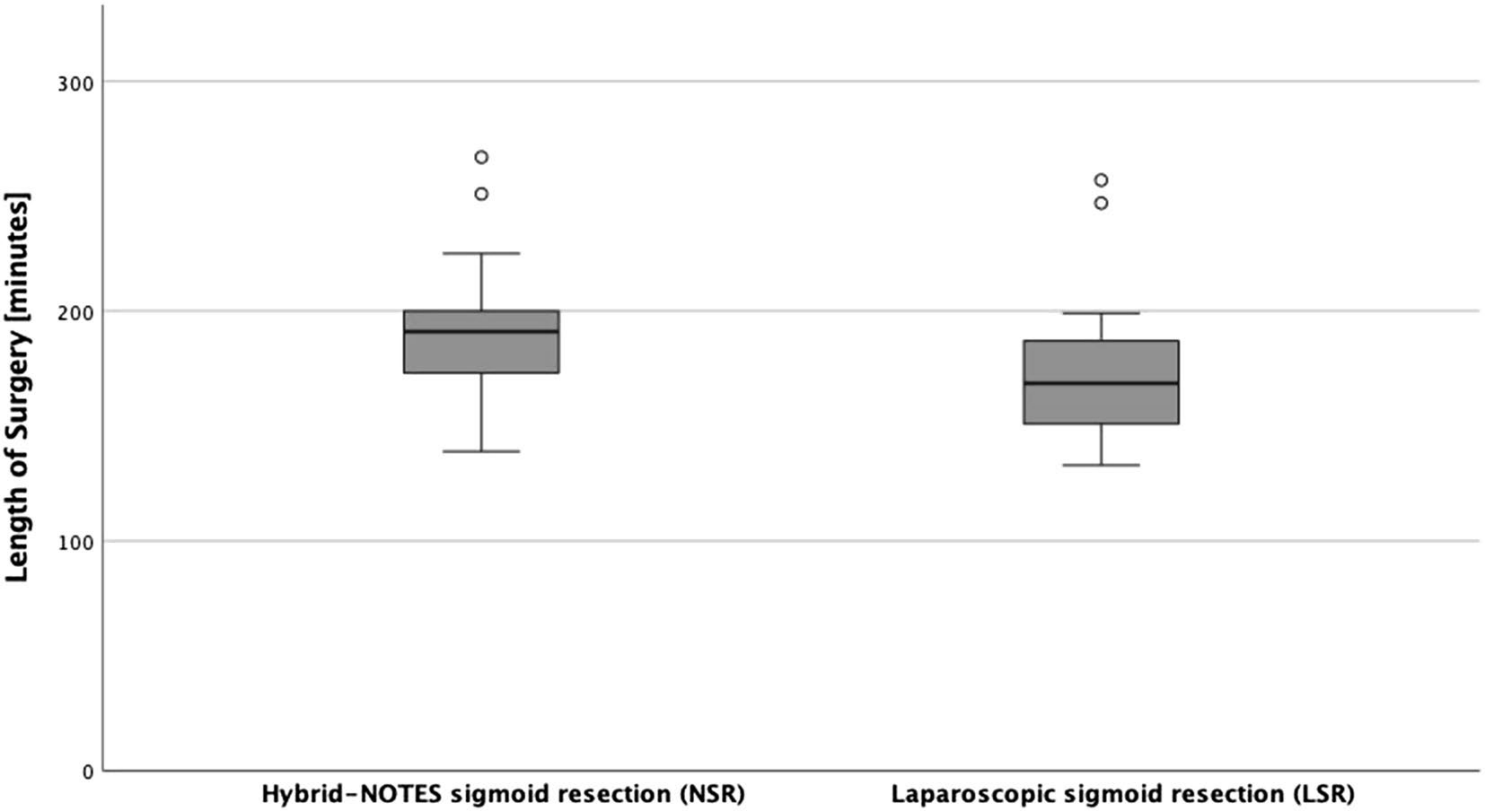
Length of surgery.

In five patients of the NSR group, a further surgical procedure was performed in addition to the sigmoid resection (one excision of a leukoplakia at the labia minora, one resection of benign omentum nodes, one cholecystectomy, one extensive adhesiolysis after previous operation, and one simultaneously liver resection); in the LSR group there were two cases (one cholecystectomy and one protective ileostomy). After excluding cases with simultaneous additional procedures for the analysis, the median length of operation (191 min) for NSR patients is not significantly higher compared to 169 min for the LSR group (Fig. 2; p = 0.079). We found also great similarities in the minima of 139 and 133 min, and the maxima of 267 and 257 min, respectively, in NSR and LSR patients. Before exclusion, the median length of surgery was 195 min in the NSR group and 169 min in the LSR group which was described as significant (p = 0.029).

No conversion to another technique was necessary in either group.

**Figure 3:**
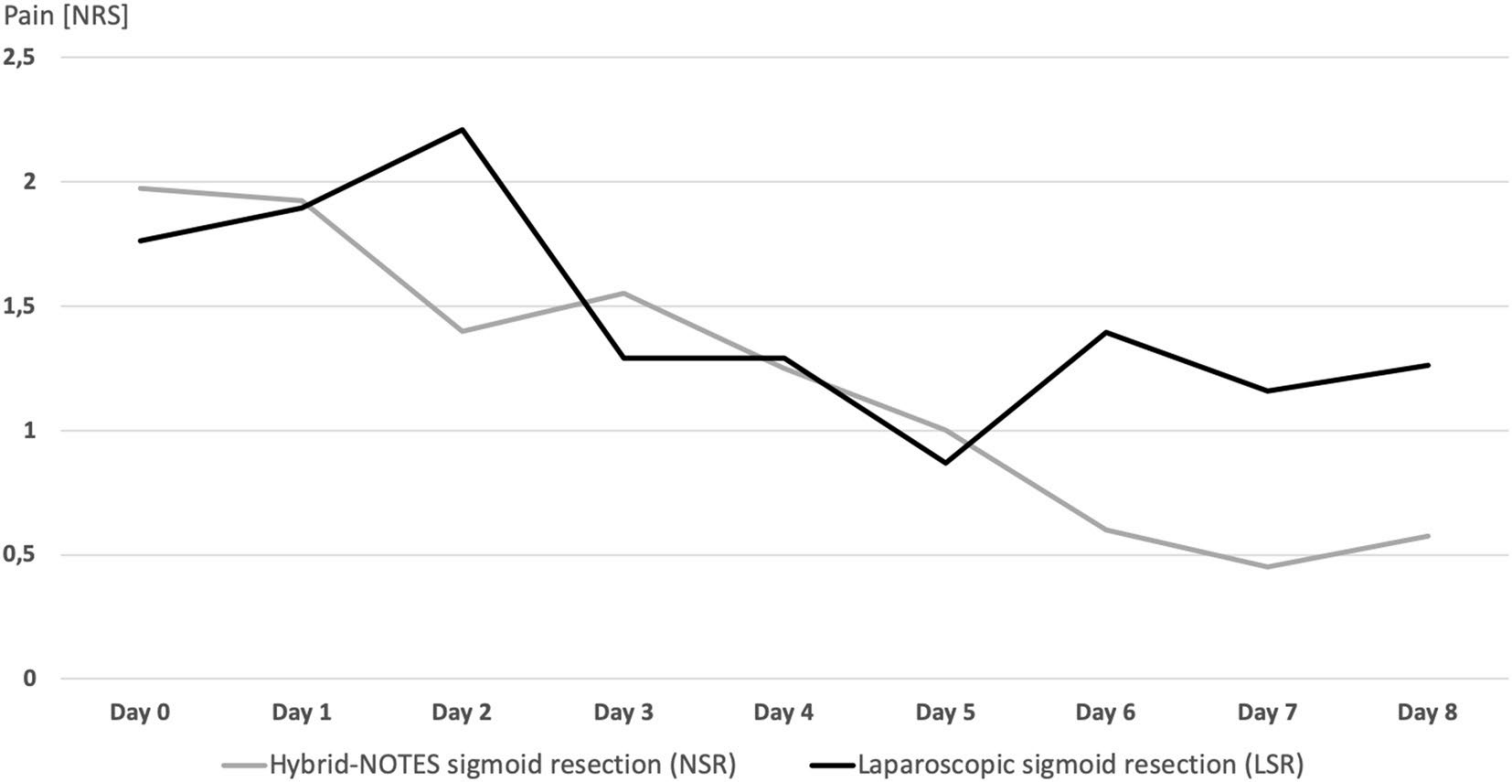
Pain intensity over time.

In the NSR group, there is a constant decrease in pain after day 3 up to the seventh postoperative day even after removal of the epidural catheter. On the eighth day, a minimal increase in pain levels from 0.5 to 0.6 is noticed. In comparison in the LSR group, pain increased on day six and remained at a higher level than in the NSR cohort (Fig. 3). If we focus on the period after epidural removal in all patients, we see that the average pain level of the LSR patients increased to 1.4 on day 6, while the pain of the NSR group decreased to 0.7. On day 7 the pain was indicated as 1.5 (LSR) compared to 0.6 (NSR), on day 8 1.3 (LSR) compared to 0.6 (NSR).

Regarding the NSR group 55% (11 patients) had an epidural catheter postoperatively where as 60% (12 patients) in the LSR group had an epidural. The median stay of the epidural catheter was 4 days in the NSR group and 5 days in the LSR patients without a significant difference (NSR: 2–5 days, LSR: 3–6 days; p = 0.336).

**Figure 4:**
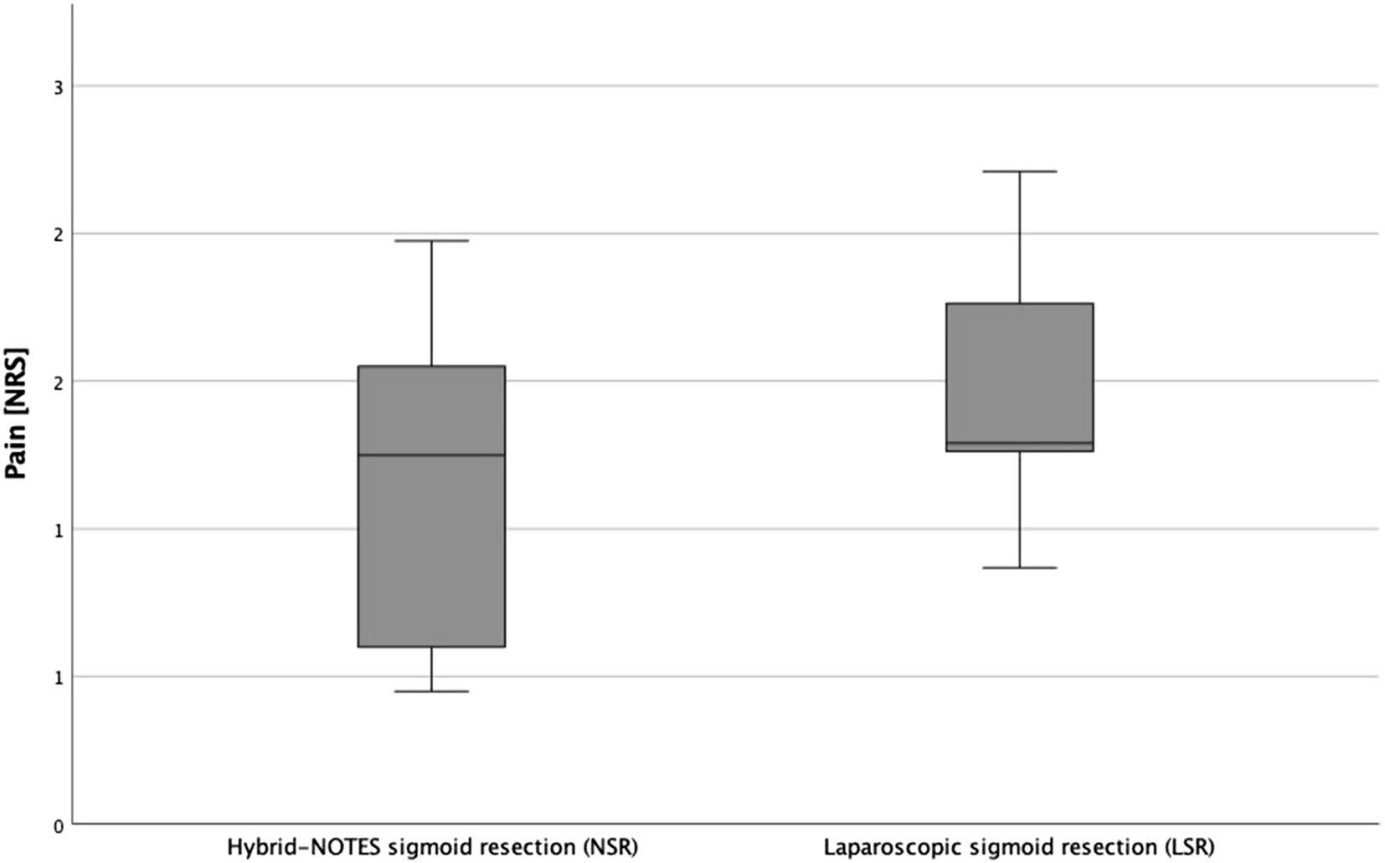
Average sum of pain intensity.

The average sum of pain intensity in both groups is compared in Fig. 4. The medians of the summarized pain values are at a similar level (LSR: 12.5; NSR: 12.0). The minimum values (LSR: 0; NSR: 0) and maximum values (LSR: 32.0; NSR: 21.5) showed no significant differences (p = 0.714).

**Figure 5:**
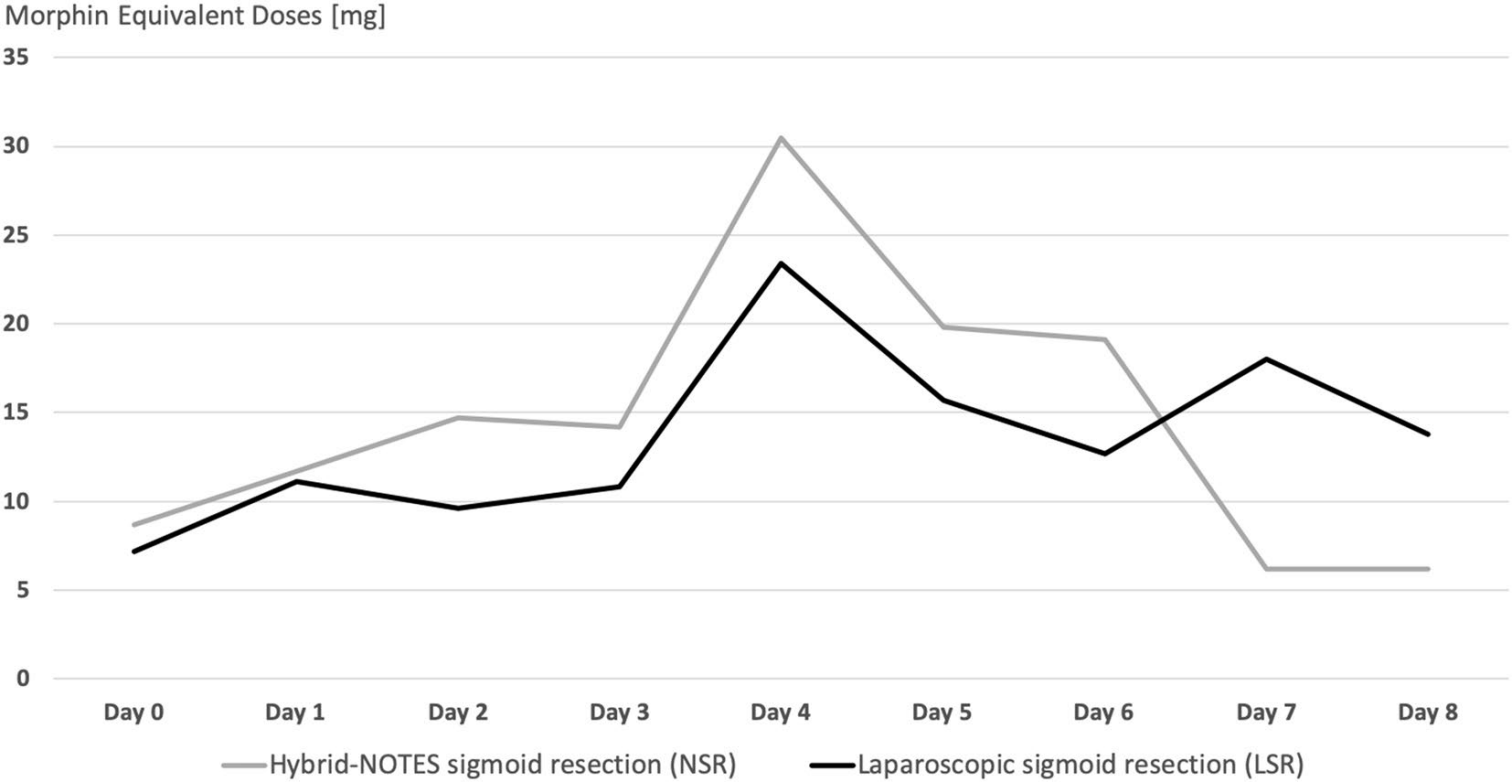
Morphine equivalent doses over time.

Figure 5 shows a similar curve in both patient cohorts regarding the morphine equivalent dose needed postoperatively. In the first three days, LSR patients required fewer opiates than patients in the NSR group but without significance (day 1: LSR M = 11.1; NSR M = 11.7 p = 0.580). From the third day onwards, the demand in both groups increased to the respective maximum of 30.5 mg on day 4 (SD = 33.4, min = 0, max = 93.3) in the NSR patients compared to 23.4 mg (SD = 25.5, min = 0.0, max = 62.5, p = 0.652) in the LSR group. Up to the eighth postoperative day, the need of morphine in the NSR patients decreased significantly, whereas the LSR patients had their lowest need on the sixth day, and it increased again until the eighth day (12.7 mg on day 6; 14.1 mg on day 8). The NSR group’s demand of morphine decreased continuously to 6.2 mg on the eighth postoperative day. The lower values of morphine of the NSR group compared to the LSR cohort on days 7 and 8 were significant (p = 0.019 and p = 0.035).

Comparing postoperative complications, there is no difference between the two surgical techniques (NSR: 3/20, LSR: 4/20; p = 0.677).

Taking the Clavien–Dindo classification of the severity of postoperative complications into account, one complication corresponding to grade I (irritation of the peroneal nerve) is found in the NSR patients. One grade II with a small abscess in the left lower abdomen postoperatively, which was treated with antibiotics and thus corresponds to and one patient with an anastomotic insufficiency (Clavien–Dindo grade IIIb).

In the LSR cohort we found three grade II complications (intestinal paralysis, secondary bleeding, acute chest pain) and one grade IIIb complication (anastomotic dehiscence).

**Figure 6:**
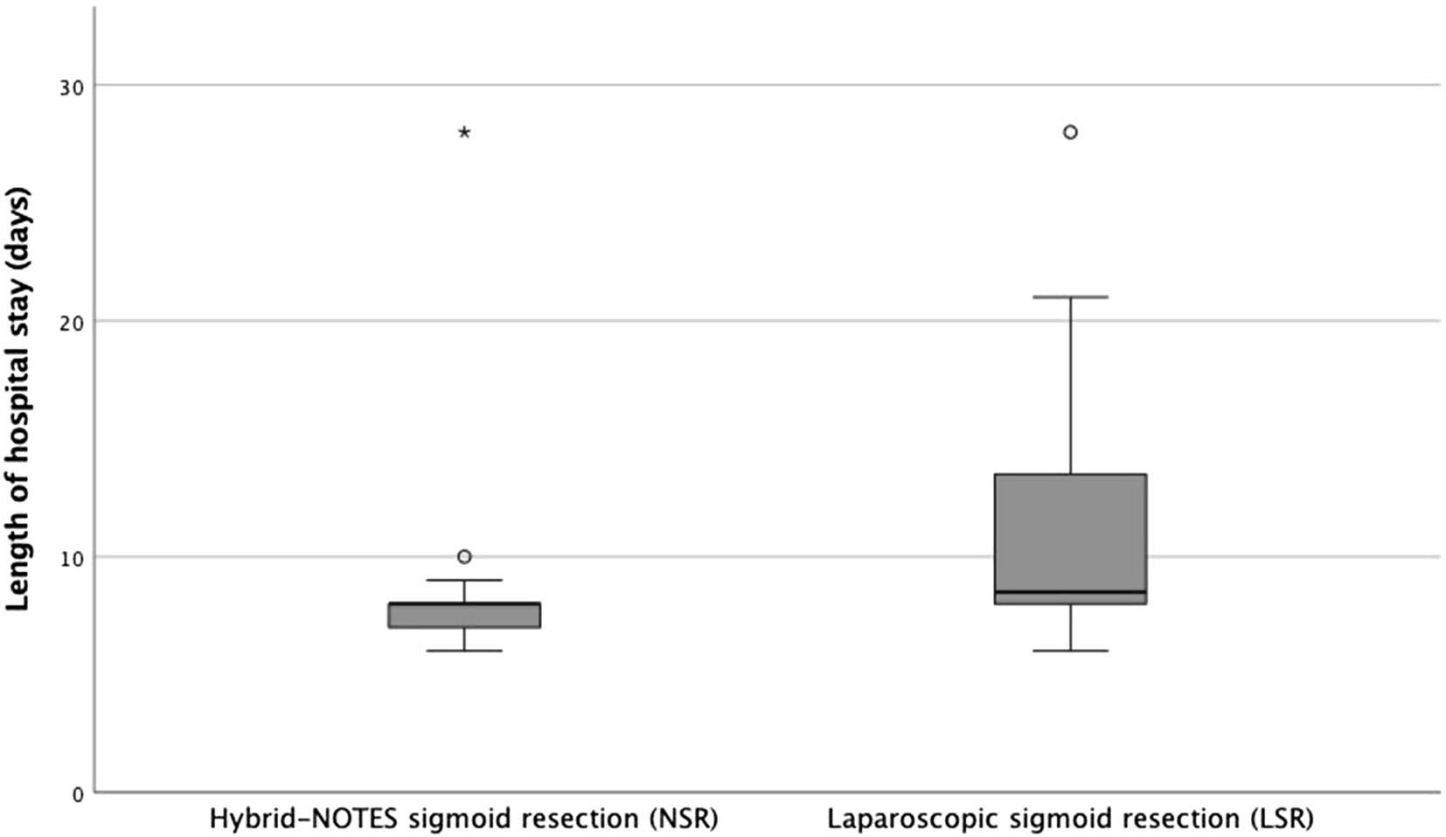
Length of hospital stay.

At last, if we look at the length of hospital stay in Fig. 6, we see a median of 8 days for NSR patients (SD = 4.6, min = 6, max = 28), and a median of 9 days for LSR patients (SD = 5.5, min = 6, max = 28). This difference is significant (p = 0.031).

## Discussion

The aim of this retrospective study was to show that transvaginal specimen removal has no major short-term disadvantages compared to the traditional laparoscopic sigmoid resection, which is considered as the surgical gold standard. According to our research, this is worldwide the first study comparing the outcome of patients after sigmoid resection including transvaginal specimen removal with the traditionally laparoscopic technique for diverticulitis. In 2014, an international multicenter randomized controlled trial comparing transvaginal hybrid NOTES versus conventionally assisted laparoscopic sigmoid resection for diverticular disease (TRANSVERSAL) was planned and registered in the German Clinical Trials Register, but till now the status of recruitment is still ‘planned’^14^.

In the following, the results of the examined parameters will be discussed.

No significant differences were found in the matching parameters (age, BMI, Hansen/Stock diverticulitis classification and ASA classification). The intraoperative parameters did not differ significantly. Postoperatively, no significant differences could be shown in the sum of the pain values of the two groups and in the duration of the epidural catheter. Also, the postoperative complication rate did not differ significantly. No conversion to another technique was necessary.

The following parameters were found to be significant in this study: on the seventh and eighth postoperative day, the morphine equivalent doses of NSR patients were significantly lower than in the LSR group, and the hospital stay of the NSR group was significantly shorter than that of the traditional laparoscopically operated patients.

The length of the operations differed significantly between the groups of all examined cases (median 195 min for the NSR group and 169 min for the LSR group). But since 25% of the NSR operations and 10% of the LSR operations had simultaneous additional procedures performed, these procedural times are only comparable to a limited extent. Therefore, the operation time was adjusted for these patients by exclusion of the patients with additional interventions. Corrected for this consideration, the median procedural time was 191 min for the NSR and 169 min for the laparoscopic sigmoid resections, which showed no significant difference. When comparing these values with the duration of the transvaginal sigmoid resection of the first 139 patients published in the German NOTES Registry (GNR) by Bulian et al.^15^, the median procedural time accounted for 127 min. A similar average procedural time of 135 min was observed in the 18 sigmoid resections performed for diverticulitis of the EURO-NOTES Clinical Registry (ECR) between April 2007 and August 2012^16^. The main difference might be that the sigmoid resections from the GNR did not only include sigmoid diverticulitis but also other diseases as surgical indications. However, 17 of the 18 sigmoid resections of the ECR were transanal surgical procedures and only 1 operation was listed as transvaginal hybrid-NOTES. Thus, the collected data is difficult to compare. The study group around Steinemann et al. evaluated NOTES surgeries in their hospital in Switzerland between 2011 and 2016. They had an average duration of 147 min in 44 transvaginal sigmoid resections for diverticular disease and were thus able to show a significant reduction in operation time compared to 173.2 min for laparoscopic sigmoid resections^17^. A recent analysis of SILS and NOTES procedures in Switzerland revealed a mean duration of the NOTES sigmoidectomy of 165.1 min^17^. In addition, NOTES sigmoidectomy was the second most frequent NOTES procedure in Switzerland with 28.0%, following cholecystectomy with 54.9%. In all these evaluations, clinic-specific differences in the surgical procedure, such as the obligatory complete mobilization of the left colon flexure in every case in our hospital, certainly play a role. This may lead to systematic differences in the procedural times and explain the longer operation time in our analysis. However, the publications described above as well as our data show that the hybrid-NOTES sigmoid resection has a comparable surgical duration and thus does not appear to be inferior to the laparoscopic surgical procedure in this respect.

Epidural analgesia plays an important role in colorectal surgery. The continuous delivery of the local anesthetic can reduce the need for opioid analgesics and at the same time achieve adequate pain reduction^18^. Typical side effects of taking systemic opioid analgesics are particularly the reduction of intestinal peristalsis causing a paralysis, which is undesirable after colorectal surgery. However, opiate-related motility disorders can be reduced by the use of an epidural catheter and a reduction in pain can be achieved^19^. Therefore, we have to bear in mind that the comparison of pain in the first four to five postoperative days with the epidural catheter is not reasonable. The median duration of the epidural was even 1 day shorter in the NSR group. This should be considered when interpreting the comparable results between the two groups in terms of postoperative pain.

Considering the results of this paper in regard to pain, there seems to be less effect in pain reduction in larger hybrid-NOTES procedures like sigmoid resections compared to less traumatic procedures such as cholecystectomies in the hybrid-NOTES technique. Bulian et al. showed significant pain reduction in hybrid NOTES cholecystectomy compared to laparoscopic gall bladder removal^20^.

One possible reason for this could be that after gall bladder removal, the primary pain is caused by the retrieval incision in the abdominal wall and therefore less severe after hybrid NOTES operations immediately after the operation. In sigmoid resections, the postoperative pain could be mainly caused by intra-abdominal manipulations during mobilization and loosening of the affected part of the intestine. Meanwhile, the retrieval incision might play a minor role in the first postoperative days. Furthermore, an epidural ensures sufficient pain reduction in the most eminent first postoperative days, so that no differences can be measured between both techniques. Realistic statements about the postoperative pain and the opioid requirement can therefore only be made after removal of an epidural. Thus, the slightly lower pain levels and significant less usage of pain medication in the days after removal of the epidural catheter could be interpreted as an advantage of NSR.

The pain level of the patients in the NSR cohort decreases continuously from the peak of the 3rd postoperative day to the 7th day and then increases slightly to an average NRS value of 0.6 on the 8th day. In the LSR group, the patients' pain increased to the maximum on the sixth day before decreasing to the eighth day, albeit with a higher level than reported by the NSR cohort. Placing these results in the context of other studies on hybrid-NOTES operations with laparoscopic interventions for colorectal diseases, a significant reduction in postoperative pain becomes apparent^21^. The mentioned meta-analysis includes nine comparative studies, of which only three studies analyzed a transvaginal specimen extraction, while the others were transanal specimen extractions. Furthermore, of these three transvaginal studies, two analyzed right hemicolectomy and one anterior resection for colorectal cancer. A recently published study found a significant pain reduction in transanal specimen extraction versus minilaparotomy after laparoscopic anterior resection for colorectal cancer^22^. However, none of the patients received an epidural catheter for postoperative pain therapy. Overall none of the studies analyzed sigmoid resections for diverticulitis.

The number of postoperative complications did not differ in both cohorts. In the NSR cohort, these were assessed as slightly less severe according to the Clavien–Dindo classification. In the analysis of the first 139 patients with colon resections from the German NOTES register, Bulian et al. showed a postoperative complication rate of 12.2% for transvaginal specimen retrieval, with 9.8% for grade III complications. Our NSR patients had a total postoperative complication rate of 15%, with 5% corresponding to grade III complications according to Clavien–Dindo. No colovaginal fistula occured. There was no difference referred to complications in the LSR group in our analysis, so we could not detect any disadvantage for the NSR regarding this aspect. Furthermore, there is no evidence that intentionally creating a fresh suture line at the posterior vaginal vault causes any problems while making a colorectal anastomosis.

Looking at the length of hospital stays in those two groups, NSR patients showed a low variance with a median of 8 days (Q1 = 7 days; Q3 = 8 days). Comparing, LSR patients were hospitalized for 9 days (Q1 = 8 days; Q3 = 14.25 days). One patient in each group was rated as an outlier due to complications (28 days each). The median hospital stay of 8 versus 9 days is very similar compared to sigmoid resections from the German NOTES register. In international comparison, the length of hospital stay seems to be quite long, which might be explained due to a different health system.

In relation to limiting factors of our study we see the low number of cases, which is due to the patient selection and the more restricted indication for surgery in diverticulitis since the update of the respective German guideline in 2014^23^. This might cause the absent of significant differences in our study e.g. in pain intensity, but also in other parameters, so that multicenter studies are needed to prove our results. The strength of our analysis especially compared to multicenter studies is the standardized procedure and postoperative treatment of all patients due to the selection of patients according to one surgeon.

## Conclusion

We found some advantages and no disadvantages of the NSR compared to LSR in patients with diverticulitis. In summary, the results suggest that the hybrid-NOTES procedure with transvaginal specimen retrieval, considering the individual risks and wishes of the patients and with the appropriate expertise of the surgeon, can be a beneficial modification of the traditional laparoscopic sigmoid resection in diverticulitis. Concerning postoperative pain and length of inpatient stay, NSR seems to indicate slight advantages compared to LSR. Due to the limited study power of our analysis we recommend further research with larger randomized trials in order to prove statistical significance and clinical relevance of these findings.
